# αv integrins: key regulators of tissue fibrosis

**DOI:** 10.1007/s00441-016-2407-9

**Published:** 2016-05-02

**Authors:** Kylie P. Conroy, Laura J. Kitto, Neil C. Henderson

**Affiliations:** MRC Centre for Inflammation Research, The Queen’s Medical Research Institute, University of Edinburgh, 47 Little France Crescent, Edinburgh, EH16 4TJ UK

**Keywords:** Integrins, Fibrosis, TGFβ, Extracellular matrix, Myofibroblasts

## Abstract

Chronic tissue injury with fibrosis results in the disruption of tissue architecture, organ dysfunction and eventual organ failure. Therefore, the development of effective anti-fibrotic therapies is urgently required. During fibrogenesis, complex interplay occurs between cellular and extracellular matrix components of the wound healing response. Integrins, a family of transmembrane cell adhesion molecules, play a key role in mediating intercellular and cell-matrix interactions. Thus, integrins provide a major node of communication between the extracellular matrix, inflammatory cells, fibroblasts and parenchymal cells and, as such, are intimately involved in the initiation, maintenance and resolution of tissue fibrosis. Modulation of members of the αv integrin family has exhibited profound effects on fibrosis in multiple organs and disease states. In this review, we discuss the current knowledge of the mechanisms of αv-integrin-mediated regulation of fibrogenesis and show that the therapeutic targeting of specific αv integrins represents a promising avenue to treat patients with a broad range of fibrotic diseases.

## Introduction

Chronic tissue injury with fibrosis results in the disruption of tissue architecture, organ dysfunction and eventual organ failure. Although fibrosis represents a massive healthcare burden worldwide, currently available therapeutic options are severely limited and organ transplantation is, in many cases, the only effective treatment for end-stage fibrotic disease. However, limited donor organ availability, high cost and co-morbidities in potential recipients mean that, on a global scale, organ transplantation can only be offered to a small percentage of patients suffering from the complications of fibrosis. The development of effective anti-fibrotic therapies is therefore essential to improving our patient care.

Fibrogenesis involves a complex interplay between the inflammatory, epithelial, myofibroblast and extracellular matrix (ECM) components of the wound healing response (Henderson and Iredale 2007; Varga and Abraham 2007). The pericellular ECM, in particular, represents a highly dynamic environment known to exert profound influences on cell behaviour. As transmembrane proteins, the integrin family of cell adhesion molecules mediates many key cell–cell and cell-matrix interactions during fibrosis. Integrins represent a major node of communication between the ECM, inflammatory cells, fibroblasts and parenchymal cells and are intimately involved in the initiation, maintenance and resolution of tissue fibrosis. Integrins are composed of non-covalent α/β heterodimers of which there are 24 known members in humans and comprise 18 different α subunits and 8 β subunits. They can translate extracellular signals, resulting in a wide range of downstream effects on cell adhesion, migration, proliferation, differentiation and apoptosis (Hynes 2002). Of particular note is the αv subunit, which forms heterodimers with the β1, β3, β5, β6 or β8 subunits. As outlined in Table 1, modulation of various members of the αv integrin family has exhibited profound effects on fibrosis in multiple organs and disease states. In this review, we will discuss the ways in which αv integrins regulate the fibrotic process and show that the therapeutic targeting of specific αv integrins represents a promising avenue for treating patients with a broad range of fibrotic diseases.

**Table 1:** Effects of αv integrin inhibition in pre-clinical models of fibrosis (*CCL*_*4*_ carbon tetrachloride, *BDL* bile duct ligation, *TAA* thioacetamide, *UUO* unilateral ureteric obstruction, *TGF* transforming growth factor, *DDC* 3,5-diethoxycarbonyl-1,4-dihydrocollidine, *IL-1β* interleukin-1β)

αv integrin subunit	Organ	Model	Method of inhibition	Summary	Reference
β1	Liver lung	CCL_4_ Bleomycin	Small molecule inhibitor, c8	Inhibition of αvβ1 significantly reduced established fibrosis in liver and lung	Reed et al. 2015
β3/β5	Liver	BDL/TAA	Small molecule inhibitor, Cilengitide	Increased hepatic collagen deposition and pro-fibrogenic gene expression	Patsenker et al. 2009
β3/β5	Lung	Bleomycin	Genetic double knockout	No protection from lung fibrosis	Atabai et al. 2009
β3	Skin	Fibrillin-1 mutation	Genetic knockout mice	Knockout of β3 rescued progression of skin stiffness and reduced collagen deposition	Gerber et al. 2015
β6	Kidney	Mouse model of Alport syndrome (Col4A3^−/−^ mice)	Function-blocking αvβ6 monoclonal antibodies β6 knockout mice	β6 knockout reduced renal fibrosis development in β6-deficient Alport mice	Hahm et al. 2007
β6	Kidney	UUO	β6 knockout mice	UUO-induced renal fibrosis is attenuated in β6-knockout mice	Ma et al. 2003
β6	Lung	TGFα over-expression	β6 function-blocking monoclonal antibody, 6.3G9 β6 knockout mice	Inhibition and genetic depletion in established fibrosis attenuated the continuation of pleural thickening and decline in lung function	Madala et al. 2014
β6	Lung	Bleomycin	β6 knockout mice.	The LAP of TGFβ is a ligand for integrin αvβ6, and it can also bind and activate TGFβ, locally regulating its function.	(Munger et al. 1999)
β6	Liver	BDL	Antibody, 3G9	Reduced acute biliary fibrosis	Wang et al. 2007
β6	Liver	BDL	Small molecule inhibitor, EMD527040	Inhibition reduced bile duct proliferation and peribiliary collagen deposition and increased fibrolytic gene expression	Patsenker et al. 2008
β6	Liver	DDC	β6 function-blocking monoclonal antibody, 3G9 and β6 knockout mice	Biliary fibrosis rescued by anti-αvβ6 antibody treatment through inhibition of progenitor cell expansion	Peng et al. 2015
β6	Lung	Radiation	β6 function-blocking monoclonal antibody, 6.3G9 and β6 knockout mice	Low dose prevented fibrogenesis; however, higher doses resulted in lung inflammation	Puthawala et al. 2008
β6	Lung	Bleomycin	β6 function-blocking monoclonal antibody, 6.3G9 and β6 knockout mice	Attenuation of lung fibrosis and reduced TGFβ activity	Horan et al. 2008
β8	Lung	IL-1β and allergen-induced lung injury	Conditional knockout of αvβ8 on fibroblasts	Inhibition of airway fibrosis in both models of lung injury	Kitamura et al. 2011

*CCL*_4_ (carbon tetrachloride), *BDL* (bile duct ligation), *TAA* (thioacetamide), *UUO* (unilateral ureteric obstruction), *TGF* (transforming growth factor), *DDC* (3,5-diethoxycarbonyl-1,4-dihydrocollidine), *IL-1β* (interleukin-1β), *LAP* (latency associated peptide

## Regulation of TGFβ activity by αv integrins

In addition to their direct effects on cellular proliferation and survival, integrins may also potentiate signals from soluble growth and survival factors. Secreted transforming growth factor beta (TGFβ) is a key regulator of fibrosis in multiple organs (Ignotz and Massagué 1986; Roberts et al. 1986; Hynes 2002; Leask and Abraham 2004). The three mammalian isoforms of TGFβ are all synthesised as precursor proteins, which are then processed by proteolytic cleavage within the endoplasmic reticulum. They are subsequently assembled as a non-covalent “small latent complex” of a disulfide-linked homodimer of the mature cytokine (a short C-terminal fragment), which is encased within a disulfide-linked homodimer of a larger amino terminal fragment called the latency-associated peptide (LAP), forming the “small latent complex”. In this form, the associated LAP homodimer prevents the mature C-terminal fragment from binding to its receptors by holding it in a conformation distinct from that of the free dimer. This “small latent complex” is further modified in the endoplasmic reticulum by disulfide linkage to another family of proteins, namely latent TGFβ binding proteins, which upon secretion are themselves chemically cross-linked to the ECM, for storing and tethering TGFβ in a latent form in the extracellular space. Tissue forces such as cellular contraction disrupt the latency cage, releasing the mature dimer and enabling it to activate TGFβ receptors. Much of the regulation of TGFβ biology thus occurs at the level of extracellular activation of this stored latent complex (Gleizes et al. 1997; Munger et al. 1997).

The three isoforms of TGFβ, namely TGFβ-1, -2 and -3, appear to have overlapping functions. All mediate their effects, at least in part, through the intracellular SMAD pathway and, of the three, TGFβ1 is the most widely involved in fibrogenesis. αv integrins have been demonstrated to play a key role in the activation of latent TGFβ1 and TGFβ3 (Annes et al. 2002). Specifically, all five integrins have been shown to interact with a linear arginine-glycine-aspartic acid (RGD) motif present in the LAP, activating latent TGF-β (Munger et al. 1999; Mu et al. 2002; Asano et al. 2006; Wipff et al. 2007). Inhibition and blockade of αvβ6 and αvβ8 phenocopies all have the developmental effects of loss of TGFβ1 and 3 (Aluwihare et al. 2008), suggesting that these two integrins are required for most or all important roles of these TGFβ isoforms during development. However, the mechanisms of TGFβ activation underlying its contribution to adult tissue pathology are less well understood.

The actin cytoskeleton also plays a role in the activation of TGFβ1, as demonstrated by the inhibition of TGFβ activity following the blockade of actin polymerisation (Munger et al. 1997) or the inhibition of Rho kinase (Jenkins et al. 2006). The mechanical force generated by integrin-mediated regulation of the actin cytoskeleton is a common mechanism for activating latent TGFβ1 (Shi et al. 2011). Shi et al. (2011) reported that mere complex formation between integrin αvβ6 and the prodomain of TGFβ1 is insufficient for its release, with the activation of TGFβ1 requiring a further force-dependent unfastening of a “straitjacket” that encircles each growth factor dimer. This is attributable to the LAP prodomain altering the conformation of TGFβ1 and effectively shielding TGFβ1 from recognition by receptors.

**Fig. 1:**
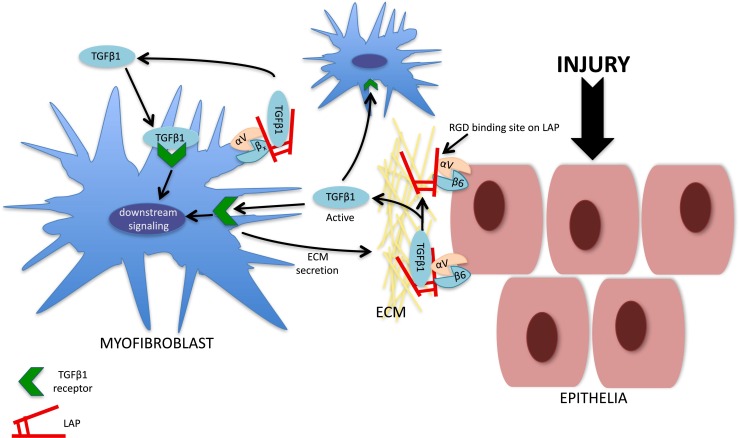
Complex interplay of αv-integrin-mediated regulation of tissue fibrosis. αv integrins (β1, β3, β5, β8) expressed on fibroblasts and αvβ6 expressed on epithelia activate transforming growth factor beta (*TGFβ*) through their interaction with a linear arginine-glycine-aspartic acid (*RGD*) binding motif present on the latency-associated peptide (*LAP*) in the extracellular matrix (*ECM*). TGFβ released from the ECM by injured epithelia might directly signal to the myofibroblast to promote further ECM production. Furthermore, αv integrins on myofibroblasts can release active TGFβ from the ECM; this TGFβ then signals in an autocrine manner to drive further ECM production by myofibroblasts

One cell type intrinsically involved in organ scarring is the myofibroblast that provides a major source of ECM proteins during fibrogenesis (Klingberg et al. 2012). The precise origin of myofibroblasts is unclear with studies indicating transdifferentiation from both local and influxing cells in response to growth factors and mechanical tension (Munger et al. 1999; Aluwihare et al. 2008; Iwaisako et al. 2014). Highly contractile cells, myofibroblasts express several αv integrins that transmit the force generated by the actin cytoskeleton to the ECM (Fig. 1). Myofibroblast αv integrins are able to liberate and thereby activate TGFβ1 deposits in the ECM via mechanical force. Further insights into this process have been provided by Klingberg et al. (2014) who demonstrated, through a series of in vitro experiments, that latent ECM-bound TGFβ1 is primed by the stiffening of the surrounding ECM, such that greater amounts are released compared with a relaxed ECM (Klingberg et al. 2014). Therefore, prior to force-mediated activation of TGFβ1, myofibroblasts actively re-organise the ECM, increasing the bioavailability of the bound latent TGFβ1 complex.

## Role of individual αv integrins during fibrogenesis

### αvβ1

Study of the function of αvβ1 during fibrogenesis has been challenging. The β1 integrin dimerises with multiple α subunits and, therefore, interrogation of the role of αvβ1 has not been possible by using standard transgenic mouse approaches. This has further been hampered by a lack of specific function-blocking antibodies or small molecule antagonists. Recently, however, a small molecule inhibitor of αvβ1, which is highly specific and potent in its activity, has been developed (Reed et al. 2015).

To develop this αvβ1 small molecule inhibitor, Reed et al. (2015) used the known base compound of αv and combined this with their previously described sulfonamidoproline moiety, which binds to the β1 subunit of α2β1 (Miller et al. 2009). The efficacy of a set of synthesised compounds in binding to individual αv integrin heterodimers and inhibiting cell adhesion was tested by using a panel of cell lines and ligands. Two compounds (c6 and c8) were found to achieve potent and specific inhibition of αvβ1 in vitro, supported by the loss of adhesion to the αvβ1 ligand, fibronectin. In cell adhesion assays in vitro, the c8 compound efficiently blocked the adhesion of fibroblasts to TGFβ1 LAP, a result that was not replicated by using antibodies to αvβ3, αvβ5, αvβ6 or αvβ8. This demonstrated that αvβ1 is the predominant fibroblastic αv integrin on fibroblasts mediating adhesion to the TGFβ1 LAP. Further in vitro characterisation, by using primary murine hepatic stellate cells and human lung fibroblasts, showed that c8 could potently and specifically inhibit TGFβ activation.

Using two murine models of fibrosis, namely carbon tetrachloride-induced liver fibrosis and bleomycin-induced lung fibrosis, Reed et al. (2015) demonstrated in vivo that the systemic delivery of c8 reduces fibrosis. Moreover, the degree of protection is comparable to that seen in a previous study, which conditionally deleted all αv integrins on myofibroblasts (Henderson et al. 2013) providing further evidence that αvβ1 is, to a large degree, responsible. Direct evidence of suppressed TGFβ signalling through reduced phosphorylation of the TGFβ intracellular signalling effector, Smad3, further suggests that this antifibrotic effect is attributable to the inhibition of αvβ1-mediated TGFβ activation.

### αvβ3 and αvβ5

Expression of αvβ3 and αvβ5 is upregulated during cardiac and skin fibrosis and both heterodimers have been reported to activate TGFβ1 signalling in vitro (Asano et al. 2004; Jenkins et al. 2006; Shi et al. 2011; Sarrazy et al. 2014). Treatment of primary cultures of scleroderma fibroblasts with anti-αvβ3 and anti-αvβ5 antibodies reduced type I procollagen expression, suggesting an anti-fibrotic effect (Asano et al. 2005, 2006; Miller et al. 2009). Furthermore, blockade of αvβ3 on culture-activated liver myofibroblasts inhibited their proliferation and increased apoptosis (Zhou et al. 2004).

However, the in vivo effects of αvβ3 and αvβ5 inhibition studied via genetic depletion and pharmacological blockade are less consistent. A β3/β5 double-knockout mouse was not protected from bleomycin-induced lung fibrosis, indicating the possible compensatory involvement of other integrins (Atabai et al. 2009). In the thioacetamide (TAA) and bile duct ligation (BDL) liver fibrosis models, αvβ3 inhibition augmented rather than reduced collagen levels (Patsenker et al. 2009). Furthermore, the αvβ3 antagonist Cilengitide failed to show any anti-fibrotic effects in two distinct models of liver fibrosis (BDL and TAA-induced injury), instead significantly increasing collagen deposition (by 31 % in BDL and 27 % in TAA) and up-regulating pro-fibrotic gene expression (Patsenker et al. 2009). Using a mouse model of stiff skin syndrome, which is caused by a fibrillin-1 mutation and leads to the development of aggressive skin fibrosis, Gerber et al. (2015) reported that β3 integrin is expressed on systemic sclerosis dermal fibroblasts.

In the context of cardiac fibrosis, αvβ3 and αvβ5 have recently been shown to compensate for each other’s function with regard to TGFβ1 activation and myofibroblast differentiation (Sarrazy et al. 2014). Through a series of in vitro studies by using transfection and short hairpin RNA to overexpress or knockdown β3 and β5 on human cardiac fibroblasts, the knockdown of one resulted in the elevated expression of the other. Conversely, the overexpression of either integrin reduced the expression of the other. Individual knock-down of β3 and β5 integrins only moderately reduced the release of active TGFβ1 and α-smooth muscle actin (α-SMA) expression, whereas the simultaneous knock-down of both integrins led to a significant reduction in TGFβ1 release and α-SMA expression compared with controls.

### αvβ6

Expression of αvβ6 integrin in the uninjured lung, liver and kidney is generally low but, in the context of inflammation and fibrosis, αvβ6 levels increase (Munger et al. 1999; Asano et al. 2005; Wang et al. 2007). Expression of αvβ6 is restricted to cells of the epithelial lineage (Breuss et al. 1995) and mediates the activation of TGFβ1 and TGFβ3 by direct binding of the integrin to the LAP (Annes et al. 2002) at epithelial surfaces (Munger et al. 1999; Shi et al. 2011). Cells expressing αvβ6 activate TGFβ1 in vitro and this can be completely inhibited by β6-blocking antibodies. Further, similar to mice homozygous for a null mutation of TGFβ1, pan-β6 integrin subunit knockout mice develop exaggerated inflammatory responses in the lungs and skin, albeit less severe than in TGFβ1 null mice (Huang et al. 1996).

In patients with fibrotic liver disease secondary to a variety of aetiologies (primary biliary cirrhosis, alcohol-induced, hepatitis B and C), integrin αvβ6 mRNA expression is increased and has been found to correlate with fibrosis stage in hepatitis C (Popov et al. 2008). Expression of αvβ6 in human chronic dermal wounds is strongly upregulated and, in the primate lung, alveolar epithelial cell αvβ6 expression is rapidly induced following lung injury (Breuss et al. 1993). In mice expressing the cytokeratin 14 promoter, driving constitutive αvβ6 expression in epidermal basal cells, spontaneous chronic skin wounds develop surrounded by fibrotic tissue (Häkkinen et al. 2004). Conversely, in aged β6-knockout mice, a significant delay in wound healing is seen compared with aged-matched controls (AlDahlawi et al. 2006), suggesting an important role in the timely resolution of injury.

In the lung, β6 null mice develop exaggerated inflammation in response to bleomycin treatment but are dramatically protected from subsequent pulmonary fibrosis (Munger et al. 1999). In β6 knockout mice treated with bleomycin, microarray analysis of the lungs identified a large group of TGFβ-inducible genes that were induced at substantially lower levels than in wild-type control mice (Kaminski et al. 2000; Atabai et al. 2009). Administration of anti-αvβ6 antibodies was able to attenuate bleomycin-induced pulmonary fibrosis with a concomitant reduction in TGFβ activity (Horan et al. 2008; Patsenker et al. 2009). Further, β6 inhibition, induced both by genetic knockout and blockade with anti-αvβ6 antibodies, was protective in radiation-induced pulmonary fibrosis (Puthawala et al. 2008; Patsenker et al. 2008). However, high doses of anti-αvβ6 monoclonal antibody resulted in lung inflammation, similar to αvβ6-null mice (Puthawala et al. 2008; Sarrazy et al. 2014). A humanised monoclonal antibody targeting αvβ6, STX-100, has been developed and is currently being evaluated in phase 2 clinical trials for the treatment of idiopathic pulmonary fibrosis.

Recently, using both pharmacological and genetic inhibition of the αvβ6 integrin, Madala et al. (2014) demonstrated that, in a TGFα model of pulmonary fibrosis, the activation of the αvβ6/TGFβ pathway has a secondary effect on pleural fibrosis. Doxycycline-induced overexpression of TGFα in lung epithelia generates progressive lung fibrosis. With this model of lung fibrosis, the authors demonstrated that the activation of the αvβ6/TGFβ pathway occurred following fibrosis development. Inhibition of αvβ6 by using function-blocking antibodies in established lung fibrosis in TGFα-overexpressing mice was able to attenuate the continuation of pleural thickening and decline in lung function. Further, in TGFα-overexpressing mice with 8 weeks of fibrosis induction, genetic ablation of β6 limited the histological and physiological pulmonary changes; however, fibrosis was still significant. This suggests that in this specific model of TGFα-induced lung fibrosis, the effective abrogation of fibrosis might require the targeting of multiple pathways in order to limit fibrogenesis and altered lung physiology.

Integrin αvβ6 has also been shown to play an important role in the progression of biliary fibrosis. In the BDL model of acute biliary fibrosis, Wang et al. (2007) demonstrated that bile duct obstruction markedly increased cholangiocyte αvβ6 expression and, moreover, that biliary fibrosis was reduced in β6 integrin null mice by 50 % compared with wild-type controls. Administration of an αvβ6-blocking antibody significantly decreased acute biliary fibrosis following BDL (Wang et al. 2007). Pharmacological inhibition of αvβ6 by using the small molecule inhibitor EMD527040 also inhibited biliary fibrosis. In BDL rats and in Mdr2 (abcb4)^−/−^ mice (a model of bile duct obstruction), treatment with EMD527040 during established fibrosis reduced bile duct proliferation and peribiliary collagen deposition by between 40 and 50 %, decreased pro-fibrotic gene expression and up-regulated fibrolytic genes (Patsenker et al. 2008). Furthermore, αvβ6 has recently been reported to be expressed on and to regulate the function of hepatic progenitor cells (Peng et al. 2015). Genetic modulation and pharmacological inhibition of αvβ6 activity during chronic liver injury strongly suppressed progenitor cell proliferation in vitro. In vivo, this led to a marked attenuation of biliary fibrogenesis and protection from fibrosis-associated liver cancer (Breuss et al. 1993; Peng et al. 2015).

In renal fibrosis, the regulatory role of αvβ6 has been investigated by using both function-blocking αvβ6 antibodies and the mouse model of Alport syndrome (Col4A3^−/−^ mice) combined with β6 integrin knockout mice (Hahm et al. 2007). Treatment with αvβ6-blocking antibody prevented the accumulation of activated fibroblasts and reduced interstitial collagen matrix deposition. Genetic ablation of β6 integrin, in a model of Alport syndrome (Col4A3^−/−^ mice), resulted in similar effects on renal fibrosis. These findings were recapitulated in the unilateral ureteric obstruction model of renal fibrosis (Ma et al. 2003). Although these data suggest a strong regulatory role for αvβ6 in renal fibrosis, the blockade of αvβ6 activity following renal transplantation has been reported to increase significantly the acute rejection of the transplanted organ (Lo et al. 2013).

### αvβ8

Mice with β8 subunit knockout exhibit vascular development defects similar to those seen in TGFβ1 null mice, with a number dying mid-gestation (Zhu et al. 2002). Of those that survive to birth, death occurs soon after from brain haemorrhage, possibly because of the loss of the developmental vascular effects of TGFβ1. Furthermore, many of these mice have a cleft palate, a prominent feature in TGFβ3 knockout mice suggesting an important role for αvβ8 in activating TGFβ3 in vivo, in addition to TGFβ1 activation in vivo (Proetzel et al. 1995; Huang et al. 1996).

Both αvβ8 and αvβ6 bind to the same RGD sequence in the LAPs of TGFβ1 and TGFβ3, although the mechanism by which they activate TGFβ1 has been shown to differ. Whereas αvβ6 requires direct cell–cell contact to activate TGFβ1, αvβ8-mediated activation requires the extracellular localisation of β8, in addition to metalloprotease (MMP) activity. Furthermore, in vitro studies have shown that αvβ8 can release active TGFβ1 into the media (Mu et al. 2002; Popov et al. 2008). Unlike αvβ6, metalloprotease inhibitors reduce αvβ8-mediated TGFβ1 activation and in vitro transfection studies have demonstrated a specific role for MT1-MMP (MMP14) in this process (Breuss et al. 1993; Mu et al. 2002). Araya and colleagues (2007) transfected primary airway fibroblasts with MMP14 short interfering RNA (siRNA), inhibiting 70 % of its surface expression leading to a marked inhibition of αvβ8-mediated activation of TGFβ. This suggests that αvβ8 activates TGFβ1 by presenting LAP complexes to cell-surface MMPs that, in turn, degrade the LAP, releasing free TGFβ1.

In chronic obstructive pulmonary disease (COPD) patients, the degree of airway wall fibrosis has been shown to correlate with αvβ8 expression on isolated airway fibroblasts (Araya et al. 2007). When incubated with interleukin-1β (IL-1β), lung fibroblasts isolated from COPD patients showed enhanced αvβ8-dependent TGFβ activation, collagen expression and pro-inflammatory gene expression compared with normal lung fibroblasts (Araya et al. 2007). Further investigation in vitro showed that the autocrine αvβ8-mediated activation of TGFβ1 contributed to the pro-fibrogenic differentiation of COPD fibroblasts, as demonstrated by using siRNA to knockdown β8 expression, leading to reduced αSMA and collagen I expression. However, this intervention inhibited only half of the total TGFβ activation when compared with a TGFβ-blocking antibody. In murine airway fibrosis, conditional deletion of lung fibroblast αvβ8 inhibited airway fibrosis in both IL-1β and ovalbumin-induced models (Kitamura et al. 2011). Furthermore, depletion of αvβ8 on cultured mouse lung fibroblasts reduced TGFβ1 activation (Kitamura et al. 2011).

In children with biliary atresia (a pediatric cholestatic disease), immunohistochemical analysis of liver biopsies has shown increased expression of αvβ8 integrin compared with healthy controls (Iordanskaia et al. 2014). Interestingly, dysregulation of MMP14 has also been reported in both human and animal models of biliary atresia (Iordanskaia et al. 2013). This suggests that the activation of TGFβ in the setting of biliary atresia may in part be mediated through both increased αvβ8 expression and altered expression of MMP14. Further studies will be required to explore this in more depth.

## Concluding remarks

The molecular pathways that regulate TGFβ signalling are attractive targets for novel anti-fibrotic therapies. However, currently available methods of pan-TGFβ inhibition target all three mammalian isoforms and, because of the critical role of TGFβ in normal tissue homeostasis, this has led to serious concerns about potential side effects. For example, given its anti-proliferative effect on most epithelial cell types, there is a risk that TGFβ1 inhibition could promote carcinogenesis. This is particularly relevant in advanced liver fibrosis, where most hepatocellular carcinomas originate from cirrhotic tissue. Additionally, because of the critical immunosuppressive role of TGFβ1 (Shull et al. 1992; Kulkarni et al. 1993; Yaswen et al. 1996), pan-TGFβ blockade can lead to excessive autoimmunity and inflammation, which could be highly deleterious in a fibrotic organ with limited functional reserve. Therefore, the inhibition of specific αv integrin subunits might allow a more refined and targeted approach to TGFβ pathway inhibition, providing the desired anti-fibrotic effects but with fewer undesirable side effects.

In recent years, it has become apparent that αv integrins play a key role in fibrosis in multiple organs. Abundant in vivo data are now available demonstrating critical regulatory roles for αv integrins expressed on multiple cell types during the fibrotic process. The component parts of tissue fibrogenesis are exquisitely complex and these studies highlight the important cross-talk between epithelia, myofibroblasts and the cells of the immune system during the development and resolution of fibrosis. Strategies to manipulate αv integrins, such as antibody blockade and small molecule inhibitors, will hopefully yield effective anti-fibrotic therapies in the future.
